# Practices and pitfalls in medication adherence in hemodialysis settings – a focus-group study of health care professionals

**DOI:** 10.1186/s12882-021-02514-8

**Published:** 2021-09-22

**Authors:** Trine Mechta Nielsen, Nina Schjerning, Gudrun Kaldan, Mads Hornum, Bo Feldt-Rasmussen, Thordis Thomsen

**Affiliations:** 1 Department of Nephrology 2132, Copenhagen University Hospital, Rigshospitalet, Copenhagen, Denmark; 2 Department of Clinical Medicine, Faculty of Health and Medical Sciences, University of Copenhagen, Copenhagen, Denmark; 3 Department of Research 7831, Copenhagen University Hospital, Rigshospitalet, Copenhagen, Denmark; 4 Herlev Acute, Critical and Emergency Science Unit - Herlev-ACES Department of Anesthesiology, Copenhagen University Hospital, Copenhagen, Denmark

**Keywords:** Attitude of Health Personnel, Hemodialysis, Medication adherence, Patient and public involvement, Qualitative research

## Abstract

**Background:**

Medication nonadherence is common among patients with hemodialysis, leading to poorer patient outcomes. Health care professionals have an important role in assessing risk of nonadherence and intervening to support adherence. The aim of this study was to explore physicians’ and nurses’ current medication adherence practices in hemodialysis settings.

**Method:**

A generic qualitative design with inductive content analysis and focus group methodology. Focus groups with health care professionals were conducted in four Nephrology Centers, representing three different regions of Denmark. An interview guide was developed in collaboration with 3 patient representatives.

**Results:**

Six focus group interviews involving a total of forty-two health care professionals were conducted. Five main categories were identified; Laboratory tests are the “gold standard” for assessing adherence, suggesting that abnormal results motivated investigation of adherence, Varying practices for supporting adherence, alluding to the impact of individual clinician priority and preference on choice of adherence interventions, Unclear allocation of roles and responsibility, specifically referring to uncertainty in the delegation of roles between physicians and nurses, Navigating time and resource limitations, intimating the resources needed to support medication adherence and Suggestions for future strategies.

**Conclusions:**

We suggest implementing systematic use of validated patient-reported outcome measures for assessing adherence and deprescribing tools to support adherence, as these instruments might identify the patients who are in most need of support and promote patient adherence to their prescribed medications. The findings also point to a need for interdisciplinary clarification of roles and responsibilities regarding medication adherence, with the aim of building a strong collaborative partnership between professions.

**Supplementary Information:**

The online version contains supplementary material available at 10.1186/s12882-021-02514-8.

## Introduction

Medications and chronic dialysis in combination constitute the primary management of patients with end stage kidney disease (ESKD). The medications used for treating concurrent chronic conditions related to ESKD are erythrocyte stimulating agents, iron supplementation, anti-hyperlipidemia, anti-hyperglycemic, anti-hypertensives and agents for derangements in calcium and phosphate metabolism [1]. For patients with ESKD adherence to medication, is therefore the cornerstone of optimal disease management.

According to the World Health Organization (WHO) adherence to medication can be defined as; the degree to which the person’s behavior corresponds with the agreed recommendations from a health care provider [2]. Nevertheless, non-adherence may be common in this patient population [1]. A recent systematic review found that patients with chronic kidney disease experienced disruption of established routines, the cost of buying medication, side effects or absence of intended therapeutic effects as barriers to their adherence. Lacking understanding of the indication for medications and not being involved in decisions concerning medication were also perceived as barriers to adherence [3]. An estimation of the prevalence of non-adherence among patients on chronic hemodialysis (HD) ranges from 12 to 99% [1], reflecting the differences in the definitions and tools used to identify non-adherence [1]. Non-adherence compromises treatment effects, increases morbidity, mortality and hospitalization, all inferring a substantial economic burden to the healthcare system [4].

According to WHO, health care professionals (HCPs) can positively impact adherence by systematically assessing risk of non-adherence and intervening to promote adherence [5].

Previous studies of HCPs’ practice for assessing adherence in hemodialysis settings primarily recruited nurses and pharmacists’ [6–8]. The studies by Ghimire et al. describe that time restraints and shortage of resource combined with insufficient training and lack of awareness challenge adherence assessment in Australia. Multidisciplinary support from pharmacists, nurses and physicians working together with active patient involvement is recommended to improving patient adherence to treatment [6–8]. Gilad et al. interviewed physicians, nurses and patients about their attitudes and approaches to medical care in a dialysis setting in Israel [9]. They found laboratory test to be used as a way of keeping patients in line which led to feelings of mistrust and irritation between HCPs and the patient [9]. To support patient’s adherence, they suggest a model of care centered on the patient [9]. The study reflects the perspective of 8 physicians, all of whom were recruited from the same site.

In Denmark, physicians and nurses are responsible for assessing adherence in hemodialysis settings. Pharmacists have no role in adherence assessment or support. We therefore wished to gain an deeper insight into physicians and nurses practice for assessing and supporting adherence to medication and their multidisciplinary collaboration in Danish hemodialysis settings.

## Methods

We conducted a generic qualitative multicenter study according to the COREQ guidelines for the conduct and reporting of qualitative research [10]. Please see supplementary material for COREQ checklist. We used inductive content analysis and focus group methodology to collect and organize data [11, 12].

### Participants selection

Participants were sampled from four Nephrology Centers, representing all geographical regions of Denmark (Jutland, Funen, Zealand). Management at each site recruited participants via purposive sampling according to the following inclusion criteria; physicians and nurses with varying levels of experience with adherence assessment practice in dialysis settings. A minimum of > 3 months practice with adherence assessment was required. Eligible participants were contacted via mail or face-to-face. All of the approached participants accepted participation. Two participants dropped out on the day of the interviews due to illness. All participants provided written informed consent prior to interviews. A total of forty-two HCP’s participated in the focus group interviews (Table 1).

**Table 1:** Overview of focus group interviews

Sites	N=	N=
	Physician	Nurse
Site 1 a (pilot)	3	
Site 1 b (pilot)		8
Site 1 c	3	3
Site 2	5	3
Site 3	4	4
Site 4	5	4

### Patient and public involvement

To strengthen the patients’ voice in our research process, we invited three patient representatives to participate in the study [13, 14]. We contacted one representative via The Danish Kidney Association and the following two representatives were contacted and recruited via internal network by the first representative. The representatives all had current or previous experience with hemodialysis treatment and represented a diversity in age, sex and education [14].

### Interviewers

The authors TMN, NS and GK conducted the focus group interviews. All interviewers had solid clinical experience within nephrology, either as a nurse or clinical nurse specialist and/or -educator. TMN and NS had established relationships with some of the participants from site 1 related to previous and present work tasks. Thus, some of the participant in site 1 knew of the project prior to attending the interviews. These participants were selected to participate in the first two pilot interviews. GK had extensive experience within focus group methodology and the moderator role. Accordingly, GK moderated the pilot interviews at site 1. TMN and NS attended the pilot interviews as trainees. After extensive reading and discussion of the moderator’s role within the team, TMN and NS conducted the rest of the interviews taking turns as moderator or observer during the interviews.

### Setting

Two pilot and four focus group interviews were conducted between February and May 2018 resulting in a total of 6 focus groups. The interviews took place in a staff or conference room near the dialysis unit at each participating site. Only the moderator team and the participant were present. Interviews lasted 60–85 min (in average 71 min). Seven of the physicians were residents in nephrology and thirteen were nephrology consultants. Twenty-two nurses participated, of whom fourteen had over 10 years of experience.

### Data collection

We initially drafted a semi-structured interview guide based on clinical experience and relevant literature [11]. The patient representatives reviewed and commented on the draft. This resulted in inclusion of questions on HCPs’ practices for managing side effects and involving patients in decisions concerning medications (Table 2). The interview guide was tested during the pilot interviews which resulted in no adjustments. None of the interviews were repeated, and each participant only attended one session. We used digital recording to collect data and transcribed the interviews verbatim. No transcripts or field notes were returned to the participants for comments. Interviews lasted between 60 and 85 min (median 71 min). Data saturation was discussed by TMN, NS and TT after the fifth interview. We jointly decided to gather more data on resident physicians’ experience with deprescribing to ensure saturation of the topic. Therefore, we conducted a sixth focus-group interview. This interview produced redundant information, resulting in the decision that data saturation had been reached.

**Table 2:** Interview guide – Questions for the focus groups

Interview guide	
• How do you assess patient’s adherence to medication?	
• What do you regard as your responsibility and role in assessing and talking to patients about adherence to medication?	
• What methods have you tried when helping patients with low adherence?	
• In what way do you involve patients in decisions such as choice of pharmaceuticals, dosage form and follow-up of side effects?	
• What facilitates or prevent you from assessing and talking to patients about adherence to medication?	
• How do you think we can optimize our efforts regarding medicine adherence?	
• What should an intervention contain?	

### Analysis

We applied inductive qualitative content analysis inspired by Elo and Kyngäs [12]. Firstly TMN, NS and TT read the interviews and field notes to become familiar with the data. TMN and NS independently coded the data using Nvivo software (Version 11, QSR International, Pty Ltd., Victoria, Australia,). After the open coding a total of 48 codes were grouped into broader higher order headings. During the analysis TMN, NS and TT held regular meetings comparing, discussing and grouping the higher order headings into sub-categories.. Sub-categories that described similar events were grouped together into categories and categories were finally grouped into five main categories. In order to enhance the credibility of the analysis we also organized a form of member check by presenting the categories to the patient representatives. During the meeting, the categories were discussed in terms of their recognizability.

## Results

We identified the following main categories through analysis of the transcribed data; Laboratory tests are relied on as the “gold standard” for assessing adherence, Varied practice for supporting adherence with 4 sub-categories, Unclear allocation of roles and responsibility, Navigating time and resource limitations and Suggestions for future strategies. Please see table 3 for selection of quotes.

**Table 3:** Selection of quotes

Categories	Quotations
Laboratory tests are the “gold standard” for assessing adherence	“If their blood tests or their blood pressure are normal, I assume that they are taking it [medication]. Or if their phosphate is ok. Then I assume they are taking the medication” (Physician site 1) “It is very much the blood tests. Whether you are a physician or nurse. The way a dialysis department works, we look at blood samples and relate them to the prescribed medications” (Nurse site 2)
Varying practices for supporting adherence	“No, it [medication adherence] is not something we regularly inquire about. I don’t think we [the department] have any focus on it” (Nurse site 1) “There is no time for you to sit down and say; Well, let’s talk about medication [..] Unless there is something wrong [..]You might say that there isn’t any real system (Nurse site 4) We can’t say that it does not take place. Because it does, of course, sometimes. But it is often a resource problem, when there is time to discuss and review the medication [..] it is not structured in a systematic way. In principle yes. But due to lack of resources, the systematic approach goes haywire. Having a dialog to uncover the extent of the problem in detail for each patient is virtually non-existent. It is sporadic and relates to individual medications” (Physician site 3)
Exploring patients’ reasons for non-adherence	“if there is a problem with the blood tests, then we talk to them about; Are you taking your phosphate binder? If it’s the one we can see is a problem, right? But I don’t talk to them about their medicine intake, if I can see that everything is running as it should and there is nothing to see in the blood tests” (Nurse site 1) “I ask the patient; How many do you take? If I say: It says here that you have to take 2 × 3 calcium tablets, then they usually just say; Yes!” (Physician Site 4)
Reviewing medication lists	“When I talk to the patient, it is important to try and explain to them why we do something about this [prescribe medication] It has to be on an informed basis that the patient make decisions” (Physician site 3) “I have made it a routine to put an arrow up or down or write “NEW” with a pen next to the new prescriptions or where I have made changes. Because when they come home and the spouse or someone else helps them with their medicine, then they can also see where I have made changes” (Physician Site 4)
Deprescribing or adding on	“As doctors we have a great responsibility to ensure that they get the medicine they need and certainly no more than that. However, there is a lot of it [prescribing medication] that runs completely unconsciously, with only modest documentation” (Physician Site 3) “Well, it is a question of clarity. If we have to get somewhere, then it is simply a matter of simplifying the patient’s medication regimens. You will be able to get really far with that. We spend a lot of time on this in the hemodialysis department. It takes time to sit down with the patient and simply get the medication lists reconciled. What are you taking? Are you taking what we think you are taking? Why don’t you take this? And then we negotiate what the patient wants to be involved in and what we need to remove in order for us to reach a common goal. It is simply a matter of getting it [medication regime] fitted so that patients can comprehend it” (Physician Site 1) “I often say; If this gives you side effects or does not work, then we have another one we can try. So that’s our way of working. And just make it clear to them, that if something bothers them, then we will try something else [medication]. Because it is of no use to us, to put them on lifelong treatment with something [medication] and that something doesn’t work for them “(Physician Site 1)
Motivational interviewing	“It [motivational interviewing] is one of the most effective tools I have been able to use so far. Instead of using our time on small talk or overwhelming the patients with too much information. You’ll find out…. Well, are they at all ready to make a change in their behavior? Where are they in their lives? Is there room for anything [information] today? Should I keep quiet or” (Nurse Site 2) If we can meet them in a way, so that they also hear from us; Well you are not the only one in the world who finds it difficult [adhering to medication regimes]. I think that it would have a very big influence on how they would tackle it” (Nurse site 1)
Unclear allocation of roles and responsibility	“It is also about how we prioritize our tasks. Because I almost always talk to my patients about fluid or elevated potassium” It’s about resources and time. It is not incorporated in the way you conduct nursing with the patient. Maybe it is also because it [talking to patients about medication] is more a physician thing - that it is just s not prioritized much among nurses” (Nurse site 1) “My patient was here today, so I should go straight in and check up on his medication intake and so on. Meanwhile also having to attend to the other three / four patients I have been assigned today? You barely have time to take care of the patients you have been assigned today. There it is, it does not happen at all!” (Nurse site 4) “But it is the law that describes who is responsible for what things. When you prescribe any medicine, you are obliged to tell how it works, side effects, how to take it, etc.” (Physician site 2) “But often it is the nurses who talk to the patients. Because they sit right beside the patients, when they start them up on dialysis. They sit and talk [..] and of course then it is often natural that you address; Do you remember to take your medicine?” (Physician site 2)
Navigating time and resource limitations	“We find that they do not take it [medication], but we do not actually find out why. Because it takes a long time” (Nurse site 2) “We actually have so much staff turnover and we are missing so many hands [nurses] at the moment. So right know there is not much control over it [who is responsible for talking to patients about medication]” (nurse site 4) “In 10 min, we have to take care of the initial problem patients come with and go through 30 pharmaceuticals; What do they take? Why and how? And so on. That is what we are asked to do, right? But then there is just something that is not adding up. [..] Yes, you try to run as fast as possible through the medication; are there any changes? and quickly ask; Do you get this medicine? but there is. You can’t manage to go through all the patient’s medications in terms of side effects, what they take, if they take one or the other. That is simply not possible” (Physician site 3) “Not that we had super much time before, but now we spend even more time at the computers. You do not have any time to talk to them [patients] about the medication, unfortunately. We don’t even have the time to tell them why you prescribe new medication anymore”. (Physician site 1)
Suggestions for future strategies	“There is no doubt about it. Those who have few resources, they are the ones who have the most difficulty. They are the ones we lose the most. Whereas the well-educated and well-off. It is clearly the ones with poor resources. It would help if we could focus on them” (Physician site 1) “We could have a check list [..] and do a monthly screening [medication adherence] [..] And then you will probably discover along the way that it is not relevant for all patients” (Nurse site 1) ” I think it is important that you regularly talk to patients about “how is it going with your medicine” and it should be scheduled how often it should be done” (Nurse site 4) “I would really like to sit down and spend an extra half hour on reviewing the medication list and make sure that they understand why they are getting the medicine and how they should take it [..]” (Physician site 1) “Social workers were amazing when they worked here. They could really help with many different things [..] Then you knew where to refer patients to [if they had difficulties paying for medication]. I think it is difficult in the framework we are subject to now, and I think we lose patients because of it” (Nurse site 1) “There is no doubt about it. If we made the regimes more individually. At least that’s my opinion. If it was the same person, they saw every time [..] So, she could continue where she left off last time. Because then she will have the opportunity to assess; Is it today I have to go all in, is the patient ready for this and that today, or should I wait until next time? The problem is that there is no overall consensus or system” (Physician site 3)

### Laboratory tests are “gold standard” for assessing adherence

Participants relied on clinical and paraclinical parameters as the primary, first-line and most valid approach to determining adherence. Both nurses and physicians acknowledged the potential pitfalls of relying solely on these measures, as they could be confounded by drug-drug and drug-food interactions, introducing a risk of misclassification. Taking this into account, both nurses and physicians categorized patients as adherent, when clinical and paraclinical tests were normal, while they suspected non-adherence when tests were abnormal. The latter motivated closer investigation of the patient’s medication adherence and initiation of supportive interventions.

### Varying practices for supporting adherence

We identified 4 main approaches to supporting adherence. The approaches were not mutually exclusive and appeared to be adopted randomly depending on the preferences, experiences, professional background and priorities of the individual HCP.

### Exploring patients’ reasons for non-adherence

When clinical and paraclinical tests indicated non-adherence, every physician, and to a lesser extent nurses, described how they engaged in investigating why patients were non-adherent. The preferred approach was interviewing with an emphasis on non-judgmental, open-ended questions such as “take me through which medications you take?”, as opposed to close-ended questions such as “have you taken this medication? This approach was adopted to avoid confrontation and forcing the patient on the defensive.

### Reviewing medication lists to promote understanding

Another common approach, especially employed by physicians but also to a small extent by nurses, was collaborative reviewing of the medication list. All physicians and some nurses saw this as an opportunity to educate patients, ensure understanding of the intended effects of the medication and the importance of adherence for achieving these effects. This included informing patients about the indications for medications, correct dosage, and side effects. Some physicians described using the patient’s medication list as a pedagogical tool for aligning medications, doses and timing of medications. Inclusion of personal hand-written comments and underlined text in the medication list aimed to endorse patients’ and relatives understanding and motivation.

### Deprescribing or adding on

Most consultants were prone to reduce complex medication regimes as a way of supporting adherence. This was done based on discussions with the individual patient about their priorities with regard to quality of life, experienced side effects and prioritization of prescribed medications. Reaching a compromise between the patient’s need for medications, acknowledgment of the patient’s values and preferences whilst prioritizing medications with the most well-established evidence-based effect was essential. Contrary to this, residents were more comfortable maintaining status quo or prescribing “add-on” medications to manage “irregularities” in clinical tests or experienced side effects. Most residents preferred to leave what they considered the more complex and potentially controversial decision of deprescribing to the consultants.

Few nurses considered deprescribing to be a potentially controversial approach, expressing concern that deprescribing might cause patients to lose faith in medicine, thereby indirectly “legalizing” non-adherence.

### Motivational interviewing

Specifically, at one site some nurses advocated motivational interviewing or elements thereof as a potential door opener specifically to complex patients with longstanding adherence challenges. When supplemented by other strategies such as dose administration aids, smartphone apps prompting patients to take their medications on time, engaging translators in consultations and enlisting the help of family members in maintaining daily medication-taking routines, motivational interviewing was perceived as a very promising intervention.

### Unclear allocation of roles and responsibilities

Nurses had varying perceptions of their role and responsibilities in medication adherence practice. Some nurses rarely engaged in conversations with patients about non-adherence, instead they viewed their responsibility as having to pass information about abnormal laboratory tests on to physicians. Other nurses took it upon themselves to investigate reasons for non-adherence, align medications together with the patient, provide up-to-date medication lists and help patients organize their medications, for example using dose administration aids.

Physicians were crystal clear about their role in prescribing, reviewing and reconciling medications with patients. They further took it for granted that nurses discussed and followed up on any medication and adherence issues with patients, ideally in relation to dialysis sessions. The nurses described that this was often not the case, however, often because other more urgent issues related to the dialysis took priority.

### Navigating time and resource limitations

The participants described an environment of care characterized by demands for efficiency combined with resource limitations. Several sites were challenged by a large turn-over of nurses. Others by implementation of new software solutions which was time-consuming and disruptive of otherwise well-established workflows both internally in the hospital and in the transfer of information to the primary care sector. At several sites, medication lists had changed format, making the reviewing process even more time-consuming. Also, recurring status consultations with nurses had been put on hold indefinitely due to implementation, resulting in consultations now taking place during dialysis in rooms with fellow patients. The frequency of physician status consultations had also been reduced in some sites. Physicians argued that they lacked enough time to complete a medication review, elicit patients’ values, experienced side effects and perceived medication barriers during consultations. In light of the complexity of the patients’ medication regimes and the fact that many patients are cognitively impaired by their kidney disease, participants experienced time restraints and resource limitations as a major barrier to comprehensive assessment and support of medication adherence.

### Suggestions for future strategies

Both nurses and physicians advocated for systematic and multidisciplinary medication adherence practices Multidisciplinary medication reviews and alignment of medications with patients were also suggested, preferably with the same HCPs attending over time to ensure continuity of care. One site specifically suggested implementing a multidisciplinary adherence team with expertise in aiding and supporting chronically ill vulnerable patients. Participants highlighted the need for social workers to untangle social and economic issues, interpreters for patients with insufficient Danish language proficiency and stronger collaboration with primary care nurses for elderly patients needing assistance in dispensing and administering medication.

## Discussion

Our findings suggest that current practices for assessing adherence relied mostly on laboratory tests, while practices for supporting adherence was dependent on the preferences, experience and prioritization of the individual clinician. Thus, a seeming lack of systematic, standardized approaches among physicians and nurses to supporting adherence was suggested. Time and resource constraints were also found to challenge comprehensive alignment of medications and treatment goals with patients. These findings resonate with prior studies and re-confirm the recommendation of more systematic medication adherence practices with active patient involvement in hemodialysis settings [6–9]. Our findings also enrich exciting knowledge by indicating that residents shy away from practicing deprescribing as a tool to support adherence. Finally our findings also indicated a lack of clarity in allocation of roles and responsibilities among involved health professions.

Laboratory tests were denoted as what we have metaphorically termed the “gold standard” for adherence assessment with sparse patient involvement if tests were characterized as normal. HCPs were nevertheless aware of the potential pitfalls of relying solely on laboratory tests, as these tests can be contaminated by drug- drug interaction and food intake. Gilad et al. also found that laboratory test had a central role in the assessment of patients’ adherence in their study [9]. Other studies have found sizeable discrepancies between physician’s estimation of patients adherence to prescribed medications and patients subjectively experienced adherence with physicians leaning towards an overestimation of patients adherence [15, 16]. Accordingly, it seems important to incorporate the patients perspective of adherence in routine care. Ghimire et al. suggested implementing checklists and validated questionnaires as a way forward [8]. We agree with this suggestion, as this approach might enhance the quality of care [17]. However, we recommend using validated patient-reported outcomes measures e.g. the 5-item Medication Adherence Report Scale (MARS-5) or the Probabilistic Medication Adherence Scale (ProMAS) [1, 18, 19]. Both have shown potential as an effective self-report screening tool and MARS-5 has already been used across a variety of health conditions, including patients with ESKD [1, 18, 19]. Both instruments may have the potential to identify the patients who are in most need of support and thereby enable more targeted allocation of resources. Our findings also indicate that current practices for supporting adherence lack consistency across professions and individual HCPs. Numerous efforts to develop effective and feasible tools to increase medication adherence have been made [20]. According to a recent review, behavioral interventions may have the potential to improve adherence to prescribed medication, although it should be noted that the certainty of the evidence was low [21]. Another review (2017) found that intervention strategies based on habit-analysis and linking medication to existing routines was associated with improved adherence outcomes [22]. A positive correlation between habit strength and adherence to medication has also been established by Badawy et al. [23]. Future research should explore the effectiveness of behavioral intervention encouraging habit formation contemplation on adherence behavior in hemodialysis settings.

Deprescribing incorporating shared decision-making was frequently applied by the consultants. According to Holmes et al. (2018), deprescribing can be defined as “the process of withdrawal of an inappropriate medication, supervised by a health care professional with the goal of managing polypharmacy and improving outcomes” [24]. In our study, consultants underlined the value of discussing priorities regarding quality of life, life expectations and experienced side effects with patients whilst also incorporating evidence to determine the optimal medication regime for the individual patient. This is supported by recent literature highlighting the fundamental importance of patient involvement and shared decision making in the deprescribing process [24–26]. However, several barriers to deprescribing have been proposed, including patient concerns about the correctness of withdrawal, fear of reliving former symptoms, adverse drug withdrawal reactions and missing out on future benefits [26–28]. According to Holmes et al. deprescribing can be seen as a preference-sensitive decision as the benefits and risks of continuing or discontinuing medication are ambiguous [24]. Engaging patients in the discussion may facilitate acceptance and adoption of deprescribing to a greater extent [29]. Nevertheless, lack of time and resources, as indicated in our findings, may challenge this [25, 28, 30]. Several tools have been developed to assist deprescribing in clinical practice [28]. However, few specifically include decision aids concerning deprescribing, and the utility of these aids in clinical practice is still unclear [31]. Decision aids for people with diabetes have been shown to reduce decisional conflict, improve knowledge, promote realistic expectations and autonomy [32]. Battistella et al. (2020) recently developed a deprescribing tool for dialysis settings consisting of medication specific algorithms including patient information tools and safety monitoring forms [29]. Implementation of deprescribing tools in dialysis settings could potentially support the complex and potentially controversial decision-making process involved in discontinuing medication [29]. It might prove particularly helpful for residents who, in our study, preferred to shy away from deprescribing. We found a lack of clarity in the allocation of roles and responsibilities in medication adherence practices, specifically, nurses differed in their perceptions. Physicians on the other hand were clear about their role and responsibilities. Lack of role clarity between HCPs regarding medication reconciliation has previously been reported as a major barrier to quality improvement in other settings [33, 34]. Formalization of roles and responsibilities in standard work-flow documents is a possible way forward [34]. Clear description of the roles and responsibilities of nurses, residents and consultants could even be empowering for nurses and residents. Moreover, it might encourage nurses who interact closely with patients several times weekly to engage more proactively in medication adherence practices during hemodialysis, as also suggested by Ghimire et al. [6]. Internationally, pharmacists have a prominent role in partnering with clinicians in adherence promoting activities [6–8]. Traditionally, pharmacists do not have a clinical role in hemodialysis settings in Denmark. In light of the challenges posed by time and resources as experienced by participants, innovation of the role of pharmacists in clinical practice might be worth considering. Not least because studies suggest that medication adherence interventions delivered by pharmacists appear significantly more effective than those delivered by nurses [22].

The strengths of our study include a comprehensive qualitative explorative multicenter approach according to the COREQ guidelines and the use of investigator- and data triangulation to enhance credibility and trustworthiness [10, 35, 36]. However, we acknowledge some study limitations. We conducted two mono-disciplinary pilot interviews and one mixed focus-groups at site 1, making this the most represented site. Participants with prior established relationship to TMN and NS were selected to participate in the first two pilot interviews moderated by GK. Analysis of the interviews from site 1 did not identify findings that differed from the other sites.

We took deliberate steps to assess data saturation during the conduct of the study. However, despite attempts to include younger resident physicians, the majority of participants had more than 10 years of experience in nephrology. We can therefore not rule out that aspects of younger clinicians’ perspectives may have been less illuminated. Throughout the conduct of the study, the research team discussed and reflected on their pre-understanding to ascertain the influence on the data collection and analysis [11, 36].

## Conclusion

This study sheds light on current medication adherence practices in Danish hemodialysis settings. Laboratory test as comprised the “gold standard” for assessing medication adherence. Medication adherence practices varied and appeared to depend on the priority and preferences of the individual HCP more than on an agreed-upon systematic, multidisciplinary approach. Participants felt challenged by time and resource restraints which compromised comprehensive medication adherence practices. Lack of clarity in professional role perceptions and allocation of interdisciplinary responsibilities for supporting non-adherent patients were additional challenges.

## Supplementary Information

**Additional file 1.**

## Data Availability

The datasets used and/or analyzed during the current study are available from the corresponding author on reasonable request.

## Abbreviations

ESKD: End Stage Kidney disease
HCPs: Health care professionals
HD: Hemodialysis
PPI: Patient and Public involvement
WHO: World Health Organization
